# Defining pain and interference recovery trajectories after acute non-catastrophic musculoskeletal trauma through growth mixture modeling

**DOI:** 10.1186/s12891-020-03621-7

**Published:** 2020-09-17

**Authors:** Joshua Y. Lee, David M. Walton, Paul Tremblay, Curtis May, Wanda Millard, James M. Elliott, Joy C. MacDermid

**Affiliations:** 1 Faculty of Health Sciences, Western University, London, ON Canada; 2 Department of Psychology, Western University, London, ON Canada; 3 Faculty of Medicine, University of British Columbia, Vancouver, BC Canada; 4 Schulich School of Medicine and Dentistry, Western University, London, ON Canada; 5 Discipline of Physiotherapy, Faculty of Health Sciences, The University of Sydney, & the Northern Sydney Local Health District; The Kolling Research Institute, St. Leonards, NSW Australia; 6 Physical Therapy and Human Movement Sciences, Feinberg School of Medicine, Northwestern University, Chicago, IL USA

## Abstract

**Background:**

Recovery trajectories support early identification of delayed recovery and can inform personalized management or phenotyping of risk profiles in patients. The objective of this study was to investigate the trajectories in pain severity and functional interference following non-catastrophic musculoskeletal (MSK) trauma in an international, mixed injury sample.

**Methods:**

A prospective longitudinal cohort (*n* = 241) was formed from patients identified within four weeks of trauma, from attendance at emergency or urgent care centres located in London, ON, Canada, or Chicago, IL, USA. Pain interference was measured via the Brief Pain Inventory (London cohort) or the Neck Disability Index (Chicago cohort). Pain severity was captured in both cohorts using the numeric pain rating scale. Growth mixture modeling and RM repeated measures ANOVA approaches identified distinct trajectories of recovery within pain interference and pain severity data.

**Results:**

For pain interference, the three trajectories were labeled accordingly: Class 1 = Rapid recovery (lowest intercept, full or near full recovery by 3 months, 32.0% of the sample); Class 2 = Delayed recovery (higher intercept, recovery by 12 months, 26.7% of the sample); Class 3 = Minimal or no recovery (higher intercept, persistently high interference scores at 12 months, 41.3% of the sample). For pain severity, the two trajectories were labeled: Class 1 = Rapid recovery (lower intercept, recovery by 3 months, 81.3% of the sample); and Class 2 = Minimal or no recovery (higher intercept, flat curve, 18.7% of the sample). The “Minimal or No Recovery” trajectory could be predicted by female sex and axial (vs. peripheral) region of trauma with 74.3% accuracy across the 3 classes for the % Interference outcome. For the Pain Severity outcome, only region (axial trauma, 81.3% accuracy) predicted the “Minimal or No Recovery” trajectory.

**Conclusions:**

These results suggest that three meaningful recovery trajectories can be identified in an international, mixed-injury sample when pain interference is the outcome, and two recovery trajectories emerge when pain severity is the outcome. Females in the sample or people who suffered axial injuries (head, neck, or low back) were more likely to be classed in poor outcome trajectories.

**Trial registration:**

National Institutes of Health - clinicaltrials.gov (NCT02711085; Retrospectively registered Mar 17, 2016).

## Background

While pain is a common feature following musculoskeletal (MSK) trauma, the phenomenon is complex, and the experience is unique to each patient. While previous models characterize pain as the direct result of tissue damage, newer models endorse multifaceted drivers recognizing the pain experience as highly subjective and influenced by interactions of biology, psychology and social influences [1]. Inconsistent relationships between clinical pain outcomes and key physiological mechanisms [2] has made management of post-trauma pain and interference difficult. Chronic pain is recognized as a distinct pathological condition [3] that disrupts daily life [4]. The incidence and prevalence of chronic pain is estimated to be nearly 20% of adults in Canada [5] and the United States [6] with large economic and social burden [7].

The inability to consistently predict or prevent the transition from acute-to-chronic is partially related to the lack of clear understanding of the mechanisms involved [8]. In addition, many prior longitudinal studies have evaluated outcomes and differences at specific time-points rather than actual trajectories. As a result, clinicians are often left to adopt a ‘wait and see’ approach to identify those patients who are not recovering, though by the time such a case arises it is too late to prevent persistent problems. We and others in the field suggest that better mechanism-based prognostic models are needed to accurately and consistently identify those at greatest risk of transitioning from acute to chronic pain [9, 10]. In this way, more targeted interventions to mitigate risk and improve distal outcomes may be attempted.

Pain prognosis as a field of study has evolved considerably over the past two decades, with emphasis added in areas such as acute whiplash-associated disorder [11] and acute low back pain [12]. However, considerable challenges persist, including i) the nature and importance of the outcomes being predicted and ii) the multitude of confounding factors influencing the value prognostic models [13]. Traditionally, pain intensity (or severity) has been the most common outcome predicted in prognostic MSK trauma research, and acute pain intensity has been a consistent predictor of those outcomes [14, 15]. For example, Panken and colleagues [16] conducted growth mixture modeling (GMM) to identify 3 trajectories that best described the trajectory of pain intensity in 622 participants with low back pain of median 5.8 weeks duration (2 to 780 weeks). Their results showed three distinct categories of recovery wherein people either had consistently low pain, consistently high pain, or showed a gradual recovery over a period of 12 months. This type of 3-class trajectory model appears to be showing consistency across other traumatic injuries, such as whiplash [17]. Outstanding questions persist including the translation of these findings to injuries affecting other parts of the body, and how choice of outcome may affect these. A better understanding of recovery trajectories will inform prognostic assessment of patients, regardless of the trauma or diagnosis. These recovery trajectories may also help to direct healthcare and theranostic (individualized treatment) resources to those who would benefit most, while reducing wasted resources for those quick to recover.

The purpose of this study was to investigate the trajectories in pain and functional interference following non-catastrophic MSK trauma in a mixed sample of both axial and peripheral non-catastrophic trauma drawn from two different countries. This was conceptualized as a first step towards a non-body-region-specific approach to prognostic clinical phenotyping of people with acute, non-catastrophic MSK trauma.

## Methods

### Participant recruitment

Data for this analysis were drawn from two longitudinal cohorts, one in London Ontario, Canada (SYMBIOME, Systematic Merging of Biology, Mental Health and Environment, clinicaltrials.gov ID no. NCT02711085) and one in Chicago, Illinois, United States (Neuromuscular Mechanisms Underlying Poor Recovery from Whiplash Injuries, clinical trials.gov ID no. NCT02157038). Eligible participants were identified by emergency or acute-care nursing or medical clinicians, all within hours to four weeks of MSK trauma. Participants were 18 to 65 years old, had to have suffered a non-catastrophic MSK injury that did not require inpatient admission or surgical correction, and could speak and understand conversational (at least grade 8) English. The London cohort included participants with non-catastrophic acute MSK injury affecting any body region, while the Chicago cohort included only those with acute whiplash-associated symptoms about the neck arising from a motor vehicle collision. Exclusion criteria were those with one or more prior motor vehicle collisions (Chicago cohort only), any metabolic systems disorders (Chicago cohort only), and any nervous system or major systemic disorders that would be expected to otherwise impair recovery independent of the trauma (e.g. active cancer, neuromuscular disease, autoimmune diseases). Co-treatment or other chronic comorbidities were captured as part of the intake and follow-up packages. Patients in the two cohorts were not matched, but cohorts were combined based on their overall study design. The recruitment environment/process, inclusion/exclusion criteria, follow-up procedures, and analysis were similar enough to facilitate a combination of databases and potentially provide more generalizable results.

After being medically cleared, interested participants provided permission for a member of the research team to describe the study, answer questions, and obtain consent to participate before leaving the hospital. Participants were provided a package of self-report questionnaires to be completed and returned within 24 h. While follow-up periods differed slightly between the two cohorts, consistent were follow-up within 2 to 4 weeks from inception, and again 3 and 12 months after injury.

### Demographics and outcomes

The constructs being captured through self-reported questionnaires were similar enough to allow meaningful pooling of the two cohorts. Both studies captured demographic and social data including age, sex, body mass index (BMI, kg/m^2^), work status, medicolegal status, and significant comorbidities (e.g. depression, existing pain conditions, etc.). The outcomes for defining recovery trajectory were pain severity using a 0–10 Numeric Pain Rating Scale (NPRS) (where 0 = no pain and 10 = extreme pain), and pain-related functional interference as measured by the Interference subscale of the Brief Pain Inventory (BPI [18], London cohort) or the Neck Disability Index (NDI [19], Chicago cohort). The BPI is one of the most widely used pain interference scales globally, [20] and has considerable evidence of validity across many clinical populations including MSK pain [21]. The NDI is one of the most widely used region-specific scales for neck disorders and captures pain interference with function on most items. The two tools share several items including work ability, sleep, and recreation, but the NDI excludes walking interference which is less relevant to those with neck pain. Both the NDI and the BPI Interference subscale have demonstrated acceptable reliability, validity, and responsiveness for capturing interference [18, 19, 21–24] and both show a strong and similar correlation with pain severity rating scales (NDI: r = 0.64 [25], BPI: r = 0.67, current database). Both can easily be converted into a percentage of the total scale range (0% = no interference, 100% = complete interference) for meaningful pooling.

Intervention between follow-up periods occurred at the discretion of the participant and their healthcare providers without influence by the research team. Type of intervention was captured in general terms (e.g. physical therapy, chiropractic, pharmaceuticals, massage therapy, work hardening) for descriptive purposes as the balance of evidence available in the field does not consistently support the superiority of any one treatment modality over another [26–29].

Ethics approval was obtained by the respective research and hospital institutional ethics boards prior to recruiting participants into the study. Participants were reimbursed up to the equivalent of $240 Canadian dollars ($175 US dollars) for expenses and time incurred during participation across all follow-up periods.

### Analysis

#### Pre-analysis

Participant demographics and baseline scores on the two outcomes were evaluated descriptively (frequencies, means, ranges). The primary (% Interference) and secondary (pain severity out of 10) outcomes were first explored for missing data and normality. Region of injury was coded according to the primary area of symptoms: presence of any head, neck or back injuries (regardless of any peripheral injuries) were classified as “axial” while those affecting the upper or lower extremities (shoulder, elbow, wrist, hip, knee, ankle) were classed as “peripheral”. As recovery was anticipated to occur in the majority of patients by 12 months, scores were square-root transformed across collection periods to reduce the skewness and kurtosis of the distribution to within acceptable limits for statistical modeling.

#### Growth mixture modeling

Maximum likelihood-based latent growth curve analysis (LGCA) using the GMM procedure in MPlus v6.12 software (Muthen & Muthen, Los Angeles USA) was conducted, following the steps of DiStefano and Kamphaus [30]. A series of models were constructed for both % Interference and Pain Severity, starting with a base single trajectory model and increasing classes until i) the model fit no longer improved substantially, ii) the estimation could not derive a mathematically valid model, or iii) one of the classes possessed fewer than 10% of participants. The fit indicators of interest were the Akaike Information Criterion (AIC) [31–33], the Bayesian Information Criterion (BIC) [31–33], and entropy [32]. While no set criteria exist for deeming an acceptable model fit [33], the cluster solution providing the lowest AIC and BIC and the highest entropy value (ideally > 0.70) that also conforms to theory is generally considered optimal [34].

An additional statistical analysis was conducted using the k-means approach, where the Lo-Mendell-Rubin Adjusted Likelihood Ratio Test (LMR-LRT) [31, 33] was used to statistically compare the fit of the k cluster solution (e.g. 3) with that of the k-1 class solution (e.g. 2). When fit did not statistically improve (*p* > 0.05) with the addition of a new class, the solution with the smaller number of classes is generally accepted for reasons of parsimony [33, 35]. All models included a quadratic (non-linear) growth function, as pre-analysis revealed the quadratic growth factor was significant in the base model. ‘Region’ (axial or peripheral) was then included as a covariate in each model to control for the effect of the different cohorts and tools. After identifying the optimal model, each participant received an assignment to the most likely trajectory (termed ‘class’) based on the highest posterior probability from the modeling procedure.

#### Missing data

Only those participants with at least two data points were included in the modeling procedure. Maximum Likelihood Estimation (MLE)-based curve estimation is useful for missing data as it uses all available data to estimate an appropriate trajectory to describe the entire sample, if the data are missing at random [36, 37]. For evidence of randomness, independent samples t-tests were conducted between those with full datasets and those with missing data. Any differences in baseline participant characteristics (i.e. age, sex, BMI, pain severity or interference) were examined for the presence of potential systematic biases in those lost to follow-up.

#### Model validation

To improve confidence in the model structures, we statistically compared the *observed* data (using all data) to the MLE-based *estimated* values from the non-region-adjusted modeling procedure using paired samples t-tests. No significant difference between observed and estimated values would indicate the predictive model was adequately accurate for estimating the data including missing values.

#### Description of trajectories

Forward-entry binary logistic regression was conducted to determine the extent to which sex, age, BMI, and region of injury (in that order) could predict trajectories using odds ratios as a standardized metric of effect size comparison across the variables. Age and BMI were dichotomized through median split (Age ≤ 38 years, BMI ≤25.09 kg/m^2^). For this analysis, the anticipated ‘worst’ (persistent problems) trajectory was coded as the state to be predicted against any other trajectories that emerged. Model fit was explored according to Peng and colleagues [38] using the Hosmer-Lemeshow (H-L) test wherein a non-significant effect indicates good fit to the data, supported by the Nagelkerke R^2^ and overall accuracy of the regression-predicted trajectory classifications. The supporting indices were needed as there were fewer than five groups to be predicted rendering the H-L test vulnerable to potentially biased estimates [38].

#### Sample size estimation

Sample size estimates for GMM are difficult to establish through traditional effect size metrics, though previous studies investigating pain recovery trajectories using GMM and ANOVA-based approaches have identified distinct classes with a medium-sized standardized difference between the classes [39]. The planned intention to conduct between-group regression analyses led to an a priori decision that a minimum of 30 participants in the smallest class was necessary. Based on work of prior authors, we anticipated the smallest class to represent about 15% of the sample, leading us to target a total sample of 200 usable datasets.

## Results

### Participant characteristics

A total of 241 participants were recruited within 28 days (4 weeks) of non-catastrophic MSK trauma, with 225 (93.4%) providing complete baseline data. Of those, 134 were from the London, Ontario sample and 97 from the Chicago, Illinois sample. The combined sample was 54.9% male, mean age of 39.7 years, BMI of 26.1 kg/m^2^, and the modal cause of injury was motor vehicle collision (50.5% of responses). Table 1 presents the remaining participant characteristics including baseline mean values on the primary outcomes. The sample was mixed between those with primarily axial (59.9%) or extremity/peripheral (40.1%) injuries. Over the 12-month period, 27% of participants reported taking over-the-counter pharmaceuticals for symptom management, 18% received physiotherapy, 10% filled and used a prescription opioid, 9% received massage therapy, and 38% received ‘other’ interventions.

**Table 1:** Participant characteristics for the entire sample (*N* = 231)

Variable (***N*** = 231)	Proportion or Mean (SD, range)
Sex (% male)	54.9%
Age (years)	39.7 (13.8, 18 to 66)
Body Mass Index (kg/m^2^)	26.1 (5.4, 14.4 to 51.5)
Cause (%)	
Motor vehicle collision	50.5%
Fall / Slip	14.2%
Hit by person or object (not MVC)	9.4%
Awkward lift or twist	8.0%
Other	17.9%
Body Region Injured (%)^1^	
Neck	52.7%
Shoulder	9.1%
Elbow	3.6%
Wrist or Hand	15.5%
Lower Back	9.1%
Hip	2.3%
Knee	8.6%
Foot or Ankle	16.4%
Employment (%)	
Full or Part-Time paid work	73.7%
Off Work (temporary)	2.6%
Not Employed for Pay	23.7%
Pain Interference (% of total score)^2^	37.6% (21.0%, 0 to 96)
Pain Severity (0–10 NRS)	4.6 (2.2, 0 to 10)

1: The total proportions will exceed 100% as participants were free to choose more than one body region

2: Disability, or functional interference was captured using the interference subscales of the Neck Disability Inventory or the Brief Pain Inventory and have been reported as a percentage of total scale score

### Growth mixture modeling

The dataset for the base model included 205 participants (axial and peripheral combined) after removing 20 participants with only a single baseline data point. Comparisons between those excluded and retained showed no difference in sex, age, BMI, or baseline pain and interference scores, supporting a random effect of missing data. Table 2 presents the results of the 2, 3, and 4-class models for both outcomes. The square-root transformed % Interference data were best described by the 3 classes after controlling for region of injury (axial or peripheral) and including a quadratic growth factor. This solution was optimal in terms of fit indicators and clinical utility with a significant LMR-LRT (LR = 60.77, *p* < 0.01) vs. the 2-class model. The three trajectories were labeled: Class 1 = Rapid recovery (lowest intercept, full or near full recovery by 3 months, 32.0% of the sample); Class 2 = Delayed recovery (higher intercept, recovery by 12 months, 26.7% of the sample); Class 3 = Minimal or no recovery (higher intercept, persistently high interference scores at 12 months, 41.3% of the sample).

**Table 2:** Fit indices for maximum likelihood-based latent growth curve models of pain severity and interference dimensions when controlling for effect of body region injured

Model	AIC	BIC	Entropy	LMR-LRT adj (p)
Pain Severity				
2-class	**2855.57**	**2909.43**	**0.86**	**103.65 (< 0.01)**
3-class	2841.72	2898.95	0.84	42.40 (0.03)
4-class	2782.97	2853.66	0.84	55.72 (0.64)
Percent Functional Interference				
2-class	2805.41	2862.72	0.73	51.55 (< 0.01)
3-class	**2752.38**	**2826.54**	**0.74**	**60.77 (< 0.01)**
4-class^1^	2734.90	2819.17	0.76	30.25 (0.27)

1: The 4-class model for % Interference would only converge when variance in both slope and quadratic growth function were constrained to zero

**Fig. 1:**
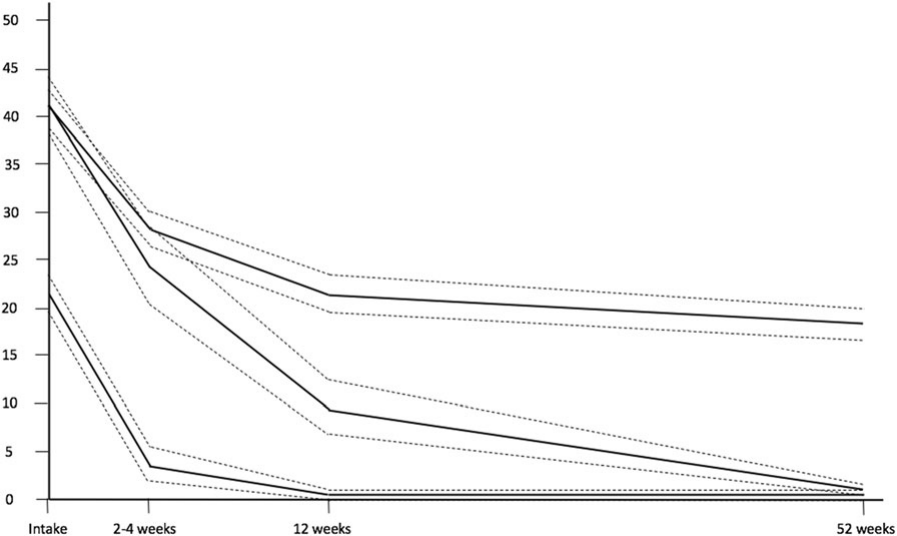
Recovery Trajectories for Pain Interference in Axial and Peripheral Injuries. Graphical representation of a 3-class LGCA model of pain interference recovery for axial and peripheral injury over a 12-month follow-up period, where dashed lines indicate 95% confidence intervals for each class. The x-axis denotes time in months (from zero at intake to 12-month follow-up) and the y-axis denotes pain interference expressed as a square-root transformed percentage. Rapid recovery (34.9%) is depicted as having a moderate intercept and rapidly declining slope. Delayed recovery (19.2%) is depicted as having a high intercept and steadily declining slope. Minimal or No Recovery (45.9%) is depicted as having a high intercept and minimally declining slope

Fit indicators for the *pain severity* outcome were optimal for a 2-trajectory quadratic growth model (AIC = 2935.51, BIC = 2983.58, Entropy = 0.79, LMR-LRT = 81.03, *p* < 0.01 vs. the single class). A 3-class model also satisfied most a priori criteria save for the proportions, where a middle 3rd “persistent moderate severity” class included only 8.3% of the sample and was therefore not retained. The two retained trajectories were labeled: Class 1 = Rapid recovery (lower intercept, recovery by 3 months, 81.3% of the sample); and Class 2 = Minimal or no recovery (higher intercept, flat curve, 18.7% of the sample). Figures 1 (% Interference) and 2 (Pain Severity) present the trajectories graphically while Table 3 provides means and 95% confidence intervals by class and time.

**Fig. 2:**
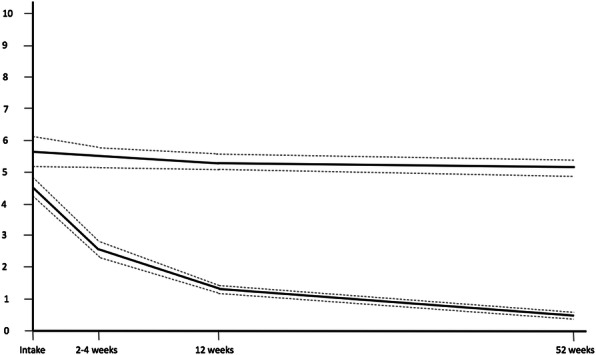
Recovery Trajectories for Pain Severity for Axial and Peripheral Injuries. Graphical representation of a 2-class LGCA model of pain severity recovery for axial and peripheral injury over a 12-month follow-up period, where dashed lines indicate 95% confidence intervals for each class. The x-axis denotes time in months (from zero at intake to 12-month follow-up) and the y-axis denotes their pain severity score out of 10. Rapid recovery (83.4%) is depicted as having a moderate intercept and steadily declining slope. Minimal or No Recovery (16.6%) is depicted as having a high intercept and minimal slope

**Table 3:** Proportions and estimated means for % Interference (Top) and Pain Severity (Bottom) trajectory classes with 95% confidence intervals (*n* = 215). Differences between classes were explored using Bonferroni-corrected post-hoc analyses for significant Class x Time interactions

% Interference							
Class	%	Baseline	1-month	3-month	6-month	Region-adjusted parameter estimates by Class	
						Slope	Quadratic
Rapid	32.0%	22.0 (19.9, 24.3)^1^	4.1 (3.1, 5.3)^1^	0.4 (0.1, 0.7)^2^	0.2 (0.0, 0.4)	−15.3	0.63
Delayed	26.7%	40.6 (37.0, 44.3)	24.8 (21.6, 28.1)	9.7 (7.8, 11.8)^2^	0.6 (0.2, 1.2)	−0.5	−0.19
Minimal	41.3%	40.7 (38.1, 43.4)	29.1 (26.6, 31.7)	21.9 (19.8, 24.2)^2^	18.1 (16.1, 20.1)^3^	−1.0	0.02
Pain Severity							
Trajectory	n	Baseline	1-month	3-month	6-month	Region-adjusted parameter estimates by Class	
						Slope	Quadratic
Rapid	82.2%	4.5 (4.2, 4.7)^2^	2.6 (2.5, 2.8)^2^	1.3 (1.2, 1.4)^2^	0.5 (0.4, 0.6)^2^	−2.0	0.23
Minimal	17.8%	5.6 (5.1, 6.1)^2^	5.5 (5.1, 5.8)^2^	5.3 (5.1, 5.6)^2^	5.1 (4.9, 5.3)^2^	−0.4	0.13

1: Mean % Interference in the *Rapid Recovery* group is significantly lower than the other two groups, with no difference between *Delayed* and *Minimal* recovery groups by virtue of overlapping confidence intervals

2: Mean % Interference / mean pain severity is significantly different across all groups

3: Mean % Interference is significantly higher in the *Minimal* recovery group than the other two groups, with no difference between the *Rapid* and *Delayed* groups

The *region* covariate (axial vs. peripheral injury) showed a significant effect on the latent class variables for both outcomes (ΔF *p* < 0.01 for both). A χ^2^ comparison of proportions revealed the effect: 92.6% (%Interference) and 91.7% (Pain Severity) of participants in the ‘Minimal or No Recovery’ trajectories were those with axial injuries, while 75.6% (% Interference) and 56.2% (Pain Severity) of those in the ‘Rapid Recovery’ trajectories described extremity injuries (χ^2^ for proportions = 83.3, p < 0.01 for % Interference; χ^2^ = 16.0, p < 0.01 for Pain Severity).

### Model validation

Paired t-tests between the available observed data and the non-adjusted, unstandardized estimated values for both outcomes revealed no significant differences at any of the 4 time points, indicating the model provided accurate estimates of the observed data.

### Class predictors

Binary logistic regression was used to explore the predictive value of the person-level variables in classifying participants into the worst (*Minimal or No Recovery*) trajectory as the index state with the other trajectory/trajectories grouped together as a single ‘recovery predicted’ trajectory. The regression models were an acceptable fit to the data (% Interference: H-L χ^2^ = 4.91, *p* = 0.77, Nagelkerke R^2^ = 0.45; Pain Severity: H-L χ^2^ = 11.89, *p* = 0.16, Nagelkerke R^2^ = 0.16). Table 4 presents the results. For % Interference as the outcome, the *Minimal or No Recovery* trajectory could be predicted by female sex and axial (vs. peripheral) region of trauma with 74.3% accuracy. For the Pain Severity outcome, only region (axial trauma) predicted the *Minimal or No Recovery* trajectory.

**Table 4:** Results of binary logistic regression for predicting class membership to the worst (Minimal or no recovery) class trajectories

	% Interference (Minimal or No Recovery)		
	B	OR (95%CI)	P
**Sex (Female)**	**0.87**	**2.39 (1.18, 4.82)**	**0.02**
Age > 38	−0.13	0.88 (0.42, 1.83)	0.73
BMI > 25.09	0.64	1.90 (0.93, 3.88)	0.08
**Axial injury**	**3.14**	**23.13 (8.43, 63.48)**	**< 0.01**
	Pain Severity (Minimal or No Recovery)		
	B	OR (95%CI)	P
Sex (Female)	0.50	1.65 (0.71, 3.83)	0.24
Age > 38	−0.17	0.84 (0.37, 1.90)	0.68
BMI > 25.09	0.44	1.55 (0.70, 3.42)	0.28
**Axial injury**	**1.93**	**6.88 (1.91, 24.69)**	**< 0.01**

*B* unstandardized beta, *OR* odds ratio. **BOLD** are variables that contributed significant predictive value to the model

## Discussion

This study has defined anticipated recovery trajectories following non-catastrophic MSK trauma in mixed geographic and body region samples. The models were created using a mixed sample of working-age adults across two different institutions, in two different countries, with injuries affecting different body regions. GMM adequately identified three trajectories of pain-related interference and two trajectories for pain severity in adult participants followed for up to 52 weeks after non-catastrophic MSK trauma. These trajectories have been labeled based on their intercept, slope, and quadratic function for use in future prognostic phenotyping work.

The trajectories for functional interference are like those derived by other authors in region-specific samples, though potentially important differences may exist. For example, Sterling and colleagues [17] identified three curvilinear classes of recovery from traumatic neck pain that could be discriminated by baseline (y-intercept) disability scores. In contrast, our model has identified a ‘Delayed Recovery’ group that entered the study with high functional interference not different from the *Minimal or No Recovery* class but had recovered by the final 12-month follow-up. Our model is more similar to that of Panken and colleagues [16] who also identified a ‘Delayed Recovery’ class in a sample of adults with low back pain, though the outcome was pain severity rather than functional interference. The middle trajectory casts some doubt on previous findings, including our own prior meta-analysis [40], suggesting that those likely to develop chronic problems can be reliably identified with higher baseline interference or pain scores. In the current analysis, those in the delayed interference recovery group would be misclassified based on baseline interference scores alone. Risk phenotyping for this group will require further exploration in future studies. This work does lend support to the notion that those who enter a longitudinal study with lower pain or interference scores are less likely to report persistent problems, and this appears to be independent of body region.

The identification of a trajectory representing over 41% of the sample that shows little or no improvement in functional scores over all time-points is concerning. However, these results are consistent with prior primary and secondary evidence that indicates approximately 50% of people following acute neck or low back injuries do not fully recover [41]. The proportion of participants in this trajectory is nearly identical to that identified by Panken and colleagues (45.2% of their sample) [16]. The Rapid Recovery group, representing 32.0% of the sample, has also been consistently identified in both neck [17, 42] and low back pain [43]. Despite some differences in shape, the 3-trajectory model has now been identified with striking consistency across clinical populations worldwide. This includes trajectories of pain and disability in a large population-level study of people with chronic pain in Canada [44], and post-operative pain in Belgium and the United States [45, 46]. Even in studies that have identified more trajectories in hip (Netherlands) [47] and low back pain (UK) [48], the existence of three stable classes represent the highest proportions with other smaller classes representing participants with fluctuating symptoms. Our a priori criterion of rejecting class structures with fewer than 10% of the sample may have masked some of these smaller groups with irregular symptoms. That said, these smaller classes may yet emerge with larger samples though we suggest that the field move with more vigor towards predicting and defining those in each individual trajectory.

Pain Severity as an outcome favoured a 2-class model. Contrary to the functional interference findings, the largest proportion for pain severity was the rapid recovery trajectory. We considered endorsing a 3-class model for consistency with interference to facilitate clinical translation. However, a third ‘moderate persistent symptoms’ trajectory could be identified using a base (unadjusted) model but included only 8.3% of the sample and was therefore rejected. Again, our trajectories appear like those of prior research. Downie and colleagues [43] followed a sample of participants with acute low back pain for 12 weeks and also identified 70.1% that showed rapid recovery. The difference in proportions between the outcomes highlights that pain severity and functional interference, while related, are distinct constructs that warrant separate investigation and may lead to different recovery status. The 2-trajectory model for Severity fits with prior work indicating that persistent pain symptoms can be predicted by pain severity at baseline [15], and that approximately 15–20% of the North American adult population will report daily chronic pain [5, 6].

The person-level variables that best predicted *Minimal or No Recovery* class membership are potentially useful for future study design. Prior work has shown that females [40] and older participants [49] are at greater risk of poor outcomes following acute neck trauma. In our sample, the odds of being in the poor interference trajectory was also approximately 2.4x greater for females than males, while age did not predict class membership for either outcome. The strongest predictor of class membership regardless of outcome however was region of injury. Those who endorsed neck or low back injuries were 23x more likely to be assigned to the poor interference trajectory and 7x more likely to be assigned to the higher pain outcome group. These results are generally in keeping with prior work showing that patients who have experienced non-catastrophic axial traumas tend to rate symptoms as more severe and more distressing than those with non-catastrophic traumas involving the extremities [50]. These results would also seem to indicate that clinicians and researchers might expect a greater proportion of poor outcomes in females reporting neck or back injuries.

A clinical summary of this work suggests that unlike what has been described in previous literature, initial symptom severity alone may not be adequate to predict long-term outcomes. Although people who rapidly recover will likely have lower levels of initial severity or disability, it is still possible to experience a full recovery despite high baseline symptoms. These individuals (as indicated by the “delayed recovery trajectory”) may recover at a slower rate, but still be fully functional by 12 months. It is not entirely clear which factors will distinguish between delayed and minimal recovery, but the data suggests that significant improvements in interference can occur by 1–3 months for those in the delayed trajectory. Close monitoring of functional status along with physical and psychosocial variables such as pre-existing psychological/pain conditions, employment status, sex, and location of trauma in the initial 3 months will be relevant for interdisciplinary treatment and the potential for early intervention. Identifying and addressing these elements as soon as possible may prevent a minimal recovery scenario.

There also appears to be more variability in pain interference compared to severity as recovery tends to occur in three (as opposed to two) different trajectories. This suggests that there may be more change associated with functional ability over the course of rehabilitation even if symptom severity remains relatively stable. Thus, physical rehabilitation may improve patient engagement by shifting the focus from symptom-related outcomes to achieving functional milestones throughout the course of treatment as this appears to be more amenable to change. These findings could provide a critical foothold in the rehabilitation process as it may influence individual expectations for recovery. Expectation management plays an essential role in determining the outcomes of MSK pain and may be more influential than specific treatments themselves [51].

Another important clinical dimension to address is the influence of ethnicity. While many of the participants did not report their ethnic group, the majority of those that did identified as Caucasian (16 out of the 19% of respondents). This made it impossible to stratify the data based on ethnicity. This has important consequences for the clinical use of this study as the results may not accurately represent the general population. In a recent review [52] investigating various dimensions of pain between ethnic groups, it was found that African-Americans tend to use more emotion-focused coping strategies compared to Caucasians. The same study also revealed some interesting differences between the US and other countries. Compared to the American population, people from Singapore seem to have a less disabling perception of pain as they rely on a more biomedical understanding of the experience. In terms of coping strategies, people from Portugal tend to use more exercise/task-oriented strategies compared to the average American.

In a qualitative study involving a multi-ethnic sample of American college students [53], it was noted that Caucasian and African American students were more likely to define pain as a negative experience compared to Asian and Hispanic students. It was also noted that cultural display rules played a role in how likely students were to express their pain to others. African American and Hispanic students felt more comfortable expressing their pain, whereas Caucasian and Asian students reported being less likely to express their pain for reasons related to stigma and embarrassment. These studies illustrate the influence of both race and ethnicity in the overall experience of pain, which would likely influence their recovery trajectory following a traumatic injury. Since many participants in our study did not report their ethnicity, we are unable to draw any legitimate conclusions about the differences between ethnic groups. Between the US and Canada, the Chicago cohort displayed a greater proportion of participants in the poor outcome groups. However, this may just be a consequence of many other factors including the location of injury rather than any sociocultural differences between the two countries.

### Limitations

The most notable risk of potential confounding in this study is the combination of two separate databases from two different countries. The participants were not matched, but both studies had very similar inclusion and exclusion criteria. While the important aspects of data collection were consistent across the two cohorts, there were obvious differences. One is the medicolegal context between the private payer system in the United States and the socialized health system in Canada. There has yet to be any compelling evidence to suggest that the rate or amount of improvement in these common MSK traumas is different between the two neighboring countries. However we acknowledge that work in other countries with less established personal injury insurance systems (e.g. Lithuania [54, 55], Greece [55, 56]) have been previously associated with different rates of chronic post-trauma neck pain compared to other western countries. Dedicated international research collaborations with standardized case definitions and outcomes are required to more fully explore the effect of personal injury insurance claims. As mentioned previously, another limitation is the paucity of data concerning ethnicity in both cohorts. Identifying meaningful patterns in the cohorts together speaks to the potential generalizability of the findings, but stratification by ethnicity would have provided key insights into the potential differences among ethnic groups. This is definitely an area for future study as it would contribute significantly to our understanding of prognosis in different populations.

The two cohorts also used different standardized patient reported outcomes for measuring functional interference, with the Chicago cohort using the NDI and the London cohort using the BPI. The two scales have never been directly compared for equality of measurement properties, though each have been independently explored against other standardized tools and shown to have similar associations [18, 19, 23, 24]. Both share two nearly identical items (sleep, work) and also include items pertaining to activity and recreation. The BPI is intended as a more generic tool, including walking, which is less relevant to those reporting neck pain only. Although we could not standardize the tools used in each database after the fact, we are confident that converting each to a percent of total scale score ensures similar constructs of “functional interference” and justifies combining the two databases for analysis. Importantly, there was no significant difference in baseline % Interference scores between the two cohorts (not shown). In addition, the inclusion of ‘region’ as a covariate in the models provided some protection against spurious findings, as the majority of those in the database with neck trauma came from the Chicago cohort. There exists a reciprocal tension between internal and external validity, and we have chosen to favor the latter through the inclusion of mixed samples, countries, and outcomes. Nevertheless, we acknowledge the effect on internal validity in doing so. It is also worth noting that the use of different, region-specific pain scales is typical within a clinical setting as functional outcomes are often tied to region-specific limitations. We have attempted to reduce these differences by ensuring that the core constructs of severity and interference were captured to some degree in both cohorts.

Another potential limitation is the sample size of the present study, although 205 is not considered small by LGCA standards. Some research groups have identified smaller trajectory classes hidden within larger samples (though these tend to represent 10% or less of the overall proportion), while other large scale analyses have similarly reported three trajectories [16, 44]. While it is possible that other trajectories comprised of smaller proportions exist in our data but were not identified, we propose that these would be rare enough to not substantively affect prognosis or treatment decisions. Finally, the use of the quadratic term made clinical and statistical sense, though a purely linear model could be identified that resulted in similar class structures with some differences in proportions assigned to each. The posterior validation steps in this study were undertaken to strengthen our confidence in the results of the quadratic models. These steps provided the added advantage of better prediction for missing data and a clear indication of recovery in the rapid class by 3-month follow-up after which the curve flattened considerably.

## Conclusion

An international mixed-injury sample of adults from with non-catastrophic MSK trauma was followed from the acute peri-injury stage through 12 months post-injury. GMM identified three meaningful trajectories of recovery when functional interference was the outcome, and two trajectories when pain severity was the outcome. Those who reported injuries affecting the axial spine (neck or low back) were more likely to be classed into the poor outcome trajectories (pain severity or interference), and females were more likely than males to be classed into the *Minimal or No Recovery* group only when functional interference was the outcome. Further exploration of person-level differences for predicting the trajectory class is a priority area for ongoing research.

## Data Availability

Datasets used and/or analyzed during the current study are available from the corresponding author on reasonable request.

## Abbreviations

MSK: Musculoskeletal
RM ANOVA: Repeated-measures Analysis of Variance
ANOVA: Analysis of Variance
SYMBIOME: Systematic Merging of Biology, Mental health, and Environment
BMI: Body mass index
NPRS: Numeric pain rating scale
BPI: Brief Pain Inventory
NDI: Neck Disability Index
LGCA: Latent Growth Curve Analysis
GMM: Growth Mixture Modeling
AIC: Akaike Information Criterion
BIC: Bayesian Information Criterion
LMR-LRT: Lo-Mendell-Rubin Adjusted Likelihood Ratio Test
MLE: Maximum Likelihood Estimation
H-L: Hosmer-Lemeshow
